# Protein kinase A antagonist inhibits β-catenin nuclear translocation, c-Myc and COX-2 expression and tumor promotion in *Apc*^Min/+ ^mice

**DOI:** 10.1186/1476-4598-10-149

**Published:** 2011-12-15

**Authors:** Kristoffer W Brudvik, Jan E Paulsen, Einar M Aandahl, Borghild Roald, Kjetil Taskén

**Affiliations:** 1 Centre for Molecular Medicine Norway, Nordic EMBL Partnership and Biotechnology Centre, University of Oslo, Oslo, Norway; 2 Norwegian School of Veterinary Science, Oslo, Norway; 3 Department of Transplantation Surgery, Oslo University Hospital Rikshospitalet, Oslo, Norway; 4 Department of Pathology, Oslo University Hospital Ullevål, Oslo, Norway

**Keywords:** *Apc*^Min/+^/b-catenin, Colorectal cancer, COX-2, protein kinase A

## Abstract

**Background:**

The adenomatous polyposis coli (APC) protein is part of the destruction complex controlling proteosomal degradation of β-catenin and limiting its nuclear translocation, which is thought to play a gate-keeping role in colorectal cancer. The destruction complex is inhibited by Wnt-Frz and prostaglandin E_2 _(PGE_2_) - PI-3 kinase pathways. Recent reports show that PGE_2_-induced phosphorylation of β-catenin by protein kinase A (PKA) increases nuclear translocation indicating two mechanisms of action of PGE_2 _on β-catenin homeostasis.

**Findings:**

Treatment of *Apc*^Min/+ ^mice that spontaneously develop intestinal adenomas with a PKA antagonist (Rp-8-Br-cAMPS) selectively targeting only the latter pathway reduced tumor load, but not the number of adenomas. Immunohistochemical characterization of intestines from treated and control animals revealed that expression of β-catenin, β-catenin nuclear translocation and expression of the β-catenin target genes c-Myc and COX-2 were significantly down-regulated upon Rp-8-Br-cAMPS treatment. Parallel experiments in a human colon cancer cell line (HCT116) revealed that Rp-8-Br-cAMPS blocked PGE_2_-induced β-catenin phosphorylation and c-Myc upregulation.

**Conclusion:**

Based on our findings we suggest that PGE_2 _act through PKA to promote β-catenin nuclear translocation and tumor development in *Apc*^Min/+ ^mice *in vivo*, indicating that the direct regulatory effect of PKA on β-catenin nuclear translocation is operative in intestinal cancer.

## Findings

The adenomatous polyposis coli (*APC) *gene is thought to play a gate-keeping role in the tumor formation and progression and is the most commonly mutated gene in all colorectal cancers. In humans, *APC *mutations can be acquired (spontaneous CRC) or inherited as in the autosomal, familiar adenomatous polyposis (FAP), characterized by the formation of multiple colonic adenomatous polyps [1]. Inactivation of both *APC *alleles (*APC*^*-/-*^) is considered necessary for tumor formation. The APC protein forms a destruction complex with Axin, glycogen synthase kinase 3β (GSK3β) and casein kinase 1 (CK1) which phosphorylates β-catenin at multiple sites [2], and targets β-catenin for ubiquitination and to degradation by the proteasome system [3]. A defective APC protein leads to cytoplasmic accumulation and translocation of β-catenin to the nucleus [4]. β-catenin, originally discovered as a cadherin-binding protein, has been shown to interact with and function as a coactivator of T-cell factor/lymphocyte enhancer factor (TCF/LEF) transcription factors. Human transcription factor 4 (hTCF-4), a TCF family member that is expressed in human colonic epithelium and colon carcinoma cells, transactivates transcription only when associated with β-catenin [5]. The result is expression and production of mitogenic and survival genes including c-Myc [6], cyclin D1 [7] and cyclooxygenase-2 (COX-2) [8].

COX-2 levels are elevated in as many as 85% of human CRCs and approximately 50% of colorectal adenomas [8]. Studies have shown that COX inhibition by non-steroidal anti-inflammatory drugs (NSAIDS) or aspirin reduces the risk of CRC and may be beneficial in large population groups at risk [9]. Selective COX-2 inhibitors are also associated with a decline in the incidence of CRC and reduced mortality rate, although COX-2 inhibitors have been associated with serious cardiovascular events in this context [10]. Prostaglandin E_2 _(PGE_2_) has been shown to be an important mediator of COX-2 associated effects, and PGE_2 _levels are elevated in CRC biopsies compared with normal mucosa and even in patient blood samples [11]. Beside an anti-angiogenic effect [12], COX inhibition promotes apoptosis and alters tumor growth [13]. PGE_2 _and COX-2 over-expression also correlates with CRC risk and metastasis of CRC [14], making this pathway relevant also in follow-up after treatment of the primary cancer. Furthermore, our observations show that the PGE_2 _produced also inhibits anti-tumor immunity through the EP2 prostanoid receptor - cAMP - protein kinase A (PKA) - Csk pathway in effector T cells that inhibit T cell activation [11].

Both the Wnt-Frz and the PGE_2_-EP3 pathway acting through phosphoinositide 3-kinase (PI3K) and protein kinase B (PKB) negatively regulates the APC destruction complex that controls β-catenin proteosomal degradation. COX inhibitors are thought to reverse the inhibitory effect of PGE_2_-EP3 receptor signaling on the APC destruction complex promoting β-catenin degradation and reversing the mitogenic effects. However, homozygous deletion of the gene for the PGE_2 _receptor EP2 also reduced the number and size of colorectal polyps in a polyposis mouse model [15]. Furthermore, recent reports have shown that PKA can phosphorylate β-catenin at Ser552 [16] and Ser675 [16,17] and that the effect of β-catenin phosphorylation at the latter site is mediated by non-canonical mechanism(s) that does not involve regulation of the formation of the destruction complex. While Taurin *et al. *show that Ser675 phosphorylation promotes β-catenin interaction with the transcriptional coactivator CREB-binding protein in the nucleus and does not affect β-catenin stability and intracellular location [16], Hino *et al. *report that PKA phosphorylation of the same site stabilizes β-catenin and affects its intracellular localization [17]. These differences highlight the complexity of regulation of Wnt-β-catenin signaling and may relate to the experimental conditions and system examined. Finally, PGE_2 _has been shown to control β-catenin homeostasis in zebrafish stem cells by signaling through both the EP3 receptor to the destruction complex and through the EP2 and EP4 receptors via cAMP to PKA affecting β-catenin stability [18]. Given the importance of β-catenin as a trans-activator in CRC and the interest in COX chemoprevention, the question of whether the PGE_2_-EP2/4-cAMP-PKA pathway is also active in controlling β-catenin levels in CRC is highly relevant [19].

The *Apc*^Min/+ ^mouse is a well-established model of FAP with a germline mutation in one *APC *allele, thus increasing the probability of a double allele mutation and tumor formation. *Apc*^Min/+ ^mice develop multiple adenomas in the intestinal tract, mainly in the small intestine, at an early age which can be blocked effectively by COX inhibition through NSAIDS. Here, we asked whether perturbation of the EP2/4 but not the EP3 pathway by inhibition at the level of PKA, could affect β-catenin levels and tumor formation. We show that treatment of *Apc*^Min/+ ^mice with a PKA antagonist, Rp-8-Br-cAMPS, reduces tumor load, β-catenin levels and nuclear translocation as well as expression of β-catenin target genes in *Apc*^Min/+ ^mice *in vivo*.

### Differential effects of COX and PKA inhibition on tumor formation in *Apc*^Min/+ ^mice

**Figure 1:**
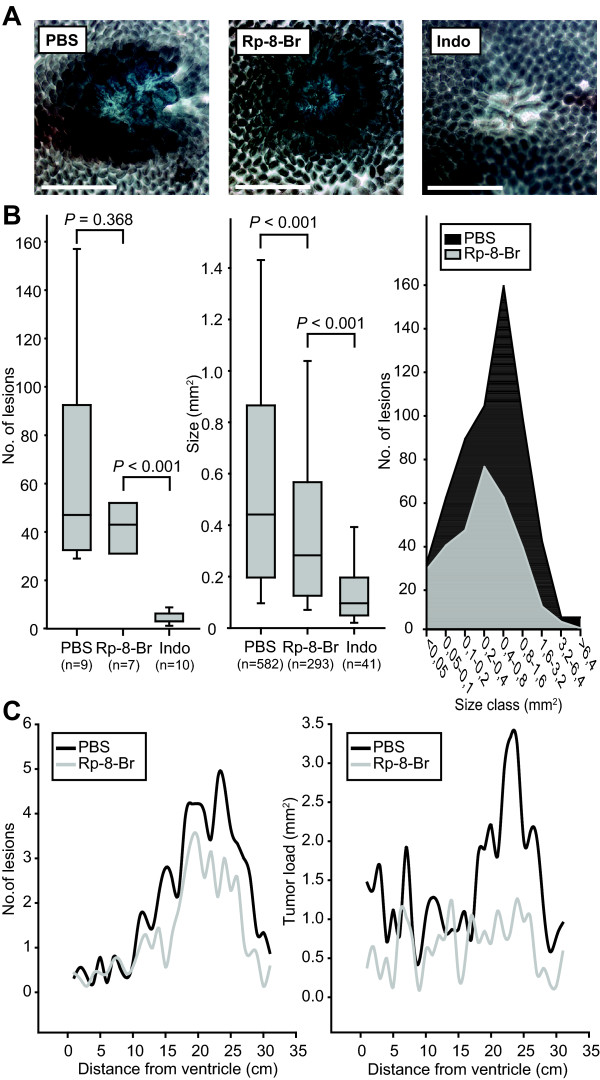
**PKA antagonist reduces tumor load in *Apc*^Min/+ ^mice**. **A**. Images of developing adenomas representative of each treatment acquired by transillumination microscopy are shown. White bars = 500 μm. **B**. Number (B; left panel) and size (B; mid panel) of spontaneously occurring tumors and distribution of tumor size in the treatment groups (B; right panel), tumor numbers/size class). Box plots with median (*horizontal line*); 25-75% (*box*) and 2.5-97.5% (*bar*) percentiles are shown for each treatment group (n = 7-10 as indicated). All tumors from the same animals were pooled when comparing tumor size distribution between the groups (n = 41-585) as indicated. Mann-Whitney Rank Sum test was used to compare the groups (SigmaPlot 11.0 (CA, USA). **C**. Number (C; left panel) and size (right panel) of spontaneously occurring tumors in the small intestine relative to the distance from the ventricle.

To more closely delineate the effect of PKA in the COX-2 - PGE_2 _pathway active in colorectal cancer, we treated *Apc*^Min/+ ^mice with the PKA antagonist Rp-8-Br-cAMPS for 6 weeks using earlier established doses (see Additional file 1, Supplementary information) and compared the result with that of treatment with the COX inhibitor indomethacin, previously shown to inhibit tumor development in the *Apc*^Min/+^model [20]. Phosphate buffered saline (PBS) was used as vehicle control for the Rp-8-Br-cAMPS. Examination revealed that indomethacin reduced the number and area of tumors in the small intestine of the *Apc*^Min/+ ^mice compared to PBS (from 47 to 3 tumors per mouse and from 0.44 mm^2 ^to 0.10 mm^2 ^tumor area; *P *< 0.001; Figure 1A, B). The PKA antagonist Rp-8-Br-cAMPS did not significantly reduce the number of adenomas (47 versus 43 tumors; *P *= 0.368, Figure 1A, B), but reduced the tumor area by 36% (from 0.44 mm^2 ^to 0.28 mm^2^; *P *< 0.001; Figure 1). Specifically, tumor load was reduced in the distal part of the small intestine (Figure 1C). The differential effect of COX inhibitor and PKA antagonist on tumor numbers and tumor size indicated to us that the mechanisms of action could be distinct and were examined in more detail in the following.

### Inhibition of PKA does not affect lymphocytic tumor infiltration or HIF-1α expression in *Apc*^Min/+ ^mice tumors

**Figure 2:**
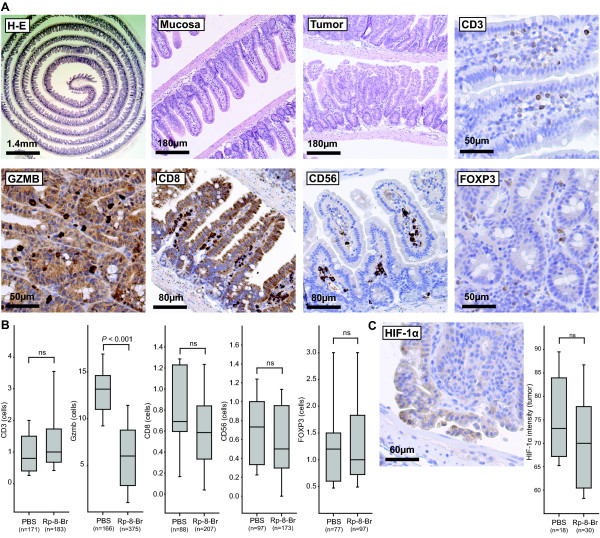
**Immunohistochemical characterization of lymphocytic infiltration and hypoxia status by HIF-1α in the *Apc*^Min/+ ^mice tumors**. **A**. Images acquired by microscopy of intestines from animals treated with PBS showing representative sections of tumor (GZMB, FOXP3) or normal small intestinal mucosa (CD3, CD8, CD56). Images with H-E stained overview, normal mucosa and tumor are also shown (top, left and middle three panels). **B**. Identifiable tumors were photographed and number of positive cells per area (n) in Rp-8-Br-cAMPS and PBS treated animals were counted using a grid. Tumors and normal tissue from all animals in each group were examined (see Additional file 2, Figure S1 for more representative images). Date are presented in Box plots with median (*horizontal line*); 25-75% (*box*) and 2.5-97.5% (*bar*) percentiles for each treatment group. **C**. The intensity of the HIF-1α staining was calculated as the mean inverse grayscale intensity of tumor area (n). A representative image from Rp-8-Br-cAMPS treated animal is shown (C; left panel) along with density staining data (C; right panel; Box plots presented as in B, n = 18-30 tumors examined). Mann-Whitney Rank Sum test was used to compare the groups in B and C; *P *< 0.05 was considered significant.

Lymphocytic tumor infiltration affects the course of human CRC where type, density and location of immune cells are shown to have higher prognostic power than the classical UICC-TNM staging [21]. Furthermore, the hypothesis of adaptive regulatory T cells (Treg) inhibiting anti-tumor immune responses has been subject to considerable interest [22]. Previously, we found that upon activation, Tregs express COX-2 and suppress effector T cells by PGE_2 _- cAMP dependent mechanisms that may be of clinical relevance in patients with CRC [11]. However, immunohistochemical characterization of small intestinal tumors from PKA antagonist Rp-8-Br-cAMPS treated animals did not reveal any significant changes in the number of CD3^+ ^T cells, CD8^+ ^cytotoxic T cells, Foxp3^+ ^Tregs or CD56^+ ^natural killer (NK) cells (Figure 2A, B and Additional file 2, Figure S1). In contrast, levels of granzyme B (GZMB), a protein expressed in the cytotoxic T lymphocytes (CD8^+ ^T cells) and NK cells, were reduced in Rp-8-Br-cAMPS treated animals which may indicate more degranulated cytotoxic cells post activation (Figure 2B). Our observations indicate that intestinal immune responses play a minor role in the development of the *Apc*^Min/+ ^mice tumor load, consistent with other observations [23].

PGE_2 _also affects angiogenesis and up-regulates vascular endothelial growth factor receptor-1 (VEGFR-1) in a human colon cancer cell line [12] whereas indomethacin inhibits the expression of VEGF and thereby angiogenesis [24]. To assess treatments effects on angiogenesis, we examined levels of the hypoxia-inducible transcription factor (HIF)-1α which regulates the expression of target genes important in angiogenesis by accumulation and translocation to the nucleus under hypoxic conditions. While apical regions of all tumors showed higher cytoplasmic intensity and nuclear staining of HIF-1α, no differences between treatment groups were observed (Figure 2C and Additional file 2, Figure S1).

### PKA antagonist treatment of *Apc*^Min/+ ^mice decreases the levels β-catenin signaling to the nucleus and of COX-2 and c-Myc expression in *Apc*^Min/+ ^mice tumors

**Figure 3:**
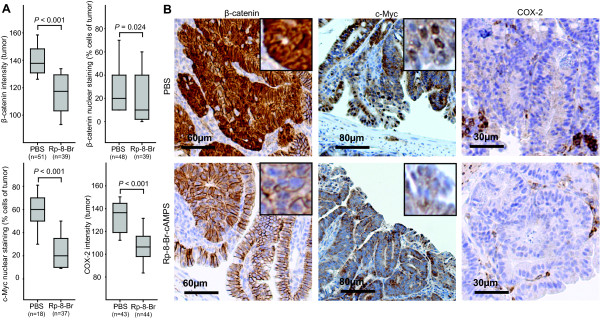
**PKA antagonist treatment of *Apc*^Min/+ ^mice decreased COX-2, β-catenin and c-Myc expression in small intestinal tumor tissue**. **A**. The intensity of the β-catenin and COX-2 staining was calculated as the mean inverse grayscale intensity of tumor area (Image J 1.43u software package). Percent β-catenin and c-Myc positive nuclei were also assessed. Data are presented as Box plots with median (*horizontal line*); 25-75% (*box*) and 2.5-97.5% (*bar*) percentiles shown for each treatment group. Median values compared with Mann-Whitney Rank Sum test when Shapiro-Wilk Normality test failed; otherwise mean values compared by Student's *t*-test. N = no. of tumors examined (tumors from every animal in each treatment group were investigated). **B**. Micrographs of representative tumors from the PBS and Rp-8-Br-cAMPS (Rp-8-Br) treated groups stained with β-catenin, c-Myc and COX-2 antibodies.

We next examined the effect of treatment on the activity of the PGE_2_- β-catenin pathway in tumor cells. As evident from image analysis of immunohistochemically stained sections, levels of β-catenin were significantly decreased in tumors from the animals treated with the PKA antagonist Rp-8-Br-cAMPS compared to tumors from the control-treated group (138 versus 117 median inverse grayscale intensity units; *P *< 0.001, Figure 3A, B) Furthermore, the median number of β-catenin positive nuclei were reduced from 20% in tumors in the control group to 10% in tumors in the Rp-8-Br-cAMPS treated group (*P *= 0.024, Figure 3A, B and Additional file 3, Figure S2). In addition, expression of the β-catenin/TCF/LEF transcription complex-regulated genes c-Myc and COX-2 were reduced in tumor cells upon treatment with the PKA antagonist Rp-8-Br-cAMPS as evident from the median number of nuclei positive for c-Myc (reduced from 60% in control to 20%; *P *< 0.001, Figure 3A, B and Additional file 3, Figure S2) and from the cytoplasmic expression levels of the COX-2 enzyme (reduced from 136 in control to 106 median inverse grayscale intensity units in the treated group; *P *< 0.001, Figure 3A, B and Additional file 3, Figure S2).

**Figure 4:**
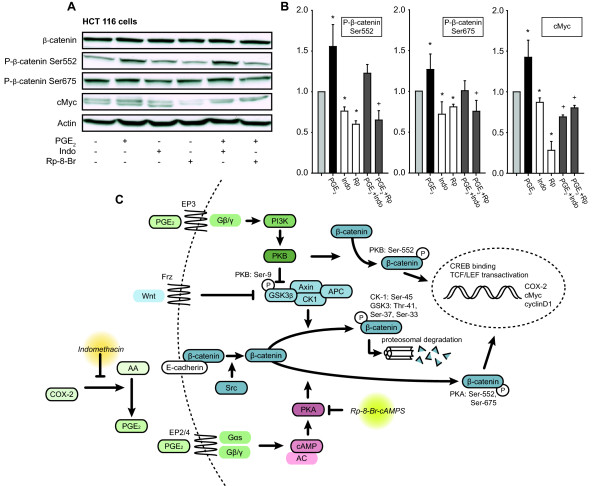
**PKA antagonist blocks β-catenin phosphorylation at Ser552 and Ser675 and down-stream c-Myc regulation in a human colon cancer cell line**. **A**. Representative immunoblots with indicated antibodies of HCT 116 colon carcinoma cells cultured in the presence of PGE_2_, indomethacin, Rp-8-Br-cAMPS or combinations as indicated. **B**. Levels of immunoreactive phospho-β-catenin Ser552 and Ser 675 and c-Myc were quantified by densitometric scanning of immunoblots. Results were normalized to total β-catenin or actin (c-Myc) levels in the same experiment. Amalgamated data from n = 3-4 experiments in individual cell cultures are shown. Median values were compared with Mann-Whitney Rank Sum test when Shapiro-Wilk Normality test failed; otherwise mean values were compared by Student's *t*-test (n = 3, left panel and n = 4, right panel) *: P < 0.05 when compared to untreated samples; +: P < 0.05 when compared to PGE2-treated samples. **C**. Schematic model of Wnt-Frz and PGE_2_-EP2-PKA pathways regulating β-catenin activity in colorectal cancer.

For further quantification of the observed effects on tumor tissue and validation of the observed effects without dilution into normal mucosa, we next looked at the regulation of β-catenin phosphorylation and c-Myc regulation in a human colonic cancer cell line, HCT 116 (Figure 4A and 4B). While treatment of HCT 116 colon carcinoma cells with PGE_2 _for 30 min (phosphorylated β-catenin) or 1 h (c-Myc) increased phosphorylation of both Ser552 and Ser675 as well as c-Myc levels, treatment with indomethacin or Rp-8-Br-cAMPS reduced levels compared to untreated sample. The latter indicates some basal prostaglandin production and PKA activation, although COX-2 levels are not sufficiently high to allow detection by Western blot in HCT 116 cells [25] (and our observations). Furthermore, the effect of exogenously added PGE_2 _on β-catenin Ser552 and Ser 675 phosphorylation could be blocked by Rp-8-Br-cAMPS but not to the same extent by indomethacin which cannot inhibit the down-stream effect of adding PGE_2 _to the cultures. In contrast, PGE_2_-mediated upregulation of c-Myc levels could be blocked in the presence of indomethacin, which may indicate that the regulation at this later time point relies more on endogenously produced PGE_2_.

Cytoplasmic β-catenin may be targeted to proteosomal degradation through the destruction complex consisting of GSK3β, Axin, CK1 and APC (Figure 4C). However, in the presence of active Wnt signaling, β-catenin accumulates in the cytosol and translocates to the nucleus to act in a mitogenic fashion by transactivation of TCF/LEF leading to expression of target genes in a cell proliferation and survival program [5]. As is well established, the Wnt-Frz pathway inhibits the destruction complex at the level of GSK3β, leading to less proteosomal degradation and more nuclear translocation and activation of β-catenin [2]. Similarly, the up-regulation of COX-2 in colorectal cancer leads to production of PGE_2 _which binds to the EP3 receptor leading to PI3K and PKB activation, phosphorylation and dissociation of GSK3β and thereby inhibition of the destruction complex [26] (Figure 4C). In zebrafish stem cells, PGE_2 _acting through an EP2/4-cAMP-PKA pathway was recently shown to induce direct phosphorylation of β-catenin, thereby stimulating its translocation to the nucleus and mitogenic effect [18]. Here, we tested whether this second pathway was providing a mitogenic drive in intestinal cancer. Using *Apc*^Min/+ ^mice with a disturbed β-catenin degradation, we specifically inhibited the PGE_2_-cAMP pathway at the level of PKA by treating mice with Rp-8-Br-cAMPS for 6 weeks (see, Figure 4C for point of action). We show that this not only reduces tumor load but also specifically inhibits β-catenin nuclear translocation and the activation of β-catenin target genes such as c-Myc and COX-2 which may indicate that the direct regulatory effect of PKA on β-catenin nuclear translocation is also operative in intestinal cancer cells. Furthermore, the fact that COX inhibitors may block the effect of PGE_2 _both in the β-catenin degradation and β-catenin nuclear translocation pathways while Rp-8-Br-cAMPS only affects the latter may explain why inhibitory effect of the PKA antagonist on tumor promotion is comparably weaker than that of indomethacin. Finally, our observation that COX inhibitor abolishes tumor numbers whereas PKA antagonist reduces tumor load but not tumor numbers may indicate that the anti-tumorigenic and anti-proliferative effects are distinct and relate to different points of action in PGE_2 _signal pathways. It is interesting to speculate that stem cells in crypt foci that give origin to adenomas may be more sensitive to regulation via the Wnt-Frz and PGE_2_-EP3 pathways than via the EP2/4-cAMP-PGE_2 _pathway whereas this balance may shift during tumor development.

## Competing interests

The authors declare that they have no competing interests.

## Authors' contributions

KWB, EMA and KT designed the experiments; KWB performed animal and WB experiments; KWB and JEP characterized intestinal lesions; KWB and BR performed and analyzed IHC images; KWB and KT wrote the manuscript with comments from all authors; all authors read and approved the final version of the manuscript.

## Supplementary Material

Additional file 1 **Supplementary information**. Materials and Methods. Reference List [27-29]. Click here for file

Additional file 2 **Figure S1**. Immunohistochemical staining with indicated antibodies of tumor and normal mucosa from small intestines from *Apc*^Min/+ ^mice treated with PBS or Rp-8-Br-cAMPS. Click here for file

Additional file 3 **Figure S2**. Immunohistochemical staining with indicated antibodies of tumor and normal mucosa from small intestines from *Apc*^Min/+ ^mice treated with PBS or Rp-8-Br-cAMPS. Click here for file

## References

[B1] Half E Bercovich D Rozen P Familial adenomatous polyposis Orphanet J Rare Dis 2009 4 22 10.1186/1750-1172-4-22 19822006 PMC2772987

[B2] Rubinfeld B Albert I Porfiri E Fiol C Munemitsu S Polakis P Binding of GSK3beta to the APC-beta-catenin complex and regulation of complex assembly Science 1996 272 1023 1026 10.1126/science.272.5264.1023 8638126

[B3] Aberle H Bauer A Stappert J Kispert A Kemler R beta-catenin is a target for the ubiquitin-proteasome pathway EMBO J 1997 16 3797 3804 10.1093/emboj/16.13.3797 9233789 PMC1170003

[B4] Städeli R Hoffmans R Basler K Transcription under the Control of Nuclear Arm/[beta]-Catenin Current Biology 2006 16 R378 R385 10.1016/j.cub.2006.04.019 16713950

[B5] Korinek V Barker N Morin PJ van Wichen D de Weger R Kinzler KW Vogelstein B Clevers H Constitutive Transcriptional Activation by a b-Catenin-Tcf Complex in APC-/- Colon Carcinoma Science 1997 275 1784 1787 10.1126/science.275.5307.1784 9065401

[B6] He TC Sparks AB Rago C Hermeking H Zawel L da Costa LT Morin PJ Vogelstein B Kinzler KW Identification of c-MYC as a Target of the APC Pathway Science 1998 281 1509 1512 9727977 10.1126/science.281.5382.1509

[B7] Tetsu O McCormick F [beta]-Catenin regulates expression of cyclin D1 in colon carcinoma cells Nature 1999 398 422 426 10.1038/18884 10201372

[B8] Eberhart CE Coffey RJ Radhika A Giardiello FM Ferrenbach S DuBois RN Up-regulation of cyclooxygenase 2 gene expression in human colorectal adenomas and adenocarcinomas Gastroenterology 1994 107 1183 1188 7926468 10.1016/0016-5085(94)90246-1

[B9] Kraus S Arber N Cancer: Do aspirin and other NSAIDs protect against colorectal cancer? Nat Rev Gastroenterol Hepatol 2011 8 125 126 10.1038/nrgastro.2010.217 21243014

[B10] Psaty BM Potter JD Risks and Benefits of Celecoxib to Prevent Recurrent Adenomas New England Journal of Medicine 2006 355 950 952 10.1056/NEJMe068158 16943408

[B11] Yaqub S Henjum K Mahic M Jahnsen FL Aandahl EM Bjornbeth BA Tasken K Regulatory T cells in colorectal cancer patients suppress anti-tumor immune activity in a COX-2 dependent manner Cancer Immunol Immunother 2008 57 813 821 10.1007/s00262-007-0417-x 17962941 PMC11030670

[B12] Fujino H Toyomura K Chen Xb Regan JW Murayama T Prostaglandin E2 regulates cellular migration via induction of vascular endothelial growth factor receptor-1 in HCA-7 human colon cancer cells Biochemical Pharmacology 2011 81 379 387 10.1016/j.bcp.2010.11.001 21070749

[B13] Sheng H Shao J Washington MK DuBois RN Prostaglandin E2 increases growth and motility of colorectal carcinoma cells J Biol Chem 2001 276 18075 18081 10.1074/jbc.M009689200 11278548

[B14] Tsujii M Kawano S DuBois RN Cyclooxygenase-2 expression in human colon cancer cells increases metastatic potential Proc Natl Acad Sci USA 1997 94 3336 3340 10.1073/pnas.94.7.3336 9096394 PMC20370

[B15] Sonoshita M Takaku K Sasaki N Sugimoto Y Ushikubi F Narumiya S Oshima M Taketo MM Acceleration of intestinal polyposis through prostaglandin receptor EP2 in Apc[Delta]716 knockout mice Nat Med 2001 7 1048 1051 10.1038/nm0901-1048 11533709

[B16] Taurin S Sandbo N Qin Y Browning D Dulin NO Phosphorylation of beta-catenin by cyclic AMP-dependent protein kinase J Biol Chem 2006 281 9971 9976 10.1074/jbc.M508778200 16476742

[B17] Hino S Tanji C Nakayama KI Kikuchi A Phosphorylation of beta-catenin by cyclic AMP-dependent protein kinase stabilizes beta-catenin through inhibition of its ubiquitination Mol Cell Biol 2005 25 9063 9072 10.1128/MCB.25.20.9063-9072.2005 16199882 PMC1265785

[B18] Goessling W North TE Loewer S Lord AM Lee S Stoick-Cooper CL Weidinger G Puder M Daley GQ Moon RT Genetic interaction of PGE2 and Wnt signaling regulates developmental specification of stem cells and regeneration Cell 2009 136 1136 1147 10.1016/j.cell.2009.01.015 19303855 PMC2692708

[B19] Buchanan FG DuBois RN Connecting COX-2 and Wnt in cancer Cancer Cell 2006 9 6 8 10.1016/j.ccr.2005.12.029 16413466

[B20] Chiu CH McEntee MF Whelan J Discordant effect of aspirin and indomethacin on intestinal tumor burden inApcMin/+ mice Prostaglandins, Leukotrienes and Essential Fatty Acids 2000 62 269 275 10.1054/plef.2000.0154 10883057

[B21] Galon J Costes A Sanchez-Cabo F Kirilovsky A Mlecnik B Lagorce-Pages C Tosolini M Camus M Berger A Wind P Type, density, and location of immune cells within human colorectal tumors predict clinical outcome Science 2006 313 1960 1964 10.1126/science.1129139 17008531

[B22] Wang RF Immune suppression by tumor-specific CD4+ regulatory T-cells in cancer Semin Cancer Biol 2006 16 73 79 10.1016/j.semcancer.2005.07.009 16140545

[B23] Kettunen HL Kettunen AS Rautonen NE Intestinal immune responses in wild-type and Apcmin/+ mouse, a model for colon cancer Cancer Res 2003 63 5136 5142 12941845

[B24] Wang HM Zhang GY Indomethacin suppresses growth of colon cancer via inhibition of angiogenesis in vivo World J Gastroenterol 2005 11 340 343 15637740 10.3748/wjg.v11.i3.340 PMC4205333

[B25] Agarwal B Swaroop P Protiva P Raj SV Shirin H Holt PR Cox-2 is needed but not sufficient for apoptosis induced by Cox-2 selective inhibitors in colon cancer cells Apoptosis 2003 8 649 654 14739610 10.1023/A:1026199929747

[B26] Castellone MD Teramoto H Williams BO Druey KM Gutkind JS Prostaglandin E2 promotes colon cancer cell growth through a Gs-axin-beta-catenin signaling axis Science 2005 310 1504 1510 10.1126/science.1116221 16293724

[B27] Andrei M Bjornstad V Langli G Romming C Klaveness J Tasken K Undheim K Stereoselective preparation of (RP)-8-hetaryladenosine-3[prime or minute],5[prime or minute]-cyclic phosphorothioic acids Org Biomol Chem 2007 5 2070 2080 10.1039/b702403g 17581650

[B28] Nayjib B Zeddou M Drion P Boniver J Tasken K Rahmouni S Moutschen M In vivo administration of a PKA type I inhibitor (Rp-8-Br-cAMPS) restores T-cell responses in retrovirus-infected mice Open Immunol J 2008 20 24

[B29] Koch T Petro A Darrabie M Opara E Effects of Esomeprazole Magnesium on Nonsteroidal Anti-Inflammatory Drug Gastropathy Digestive Diseases and Sciences 2005 50 86 93 10.1007/s10620-005-1283-z 15712643

[B30] Paulsen JE Steffensen IL Andreassen A Vikse R Alexander J Neonatal exposure to the food mutagen 2-amino-1-methyl-6-phenylimidazo[4,5-b]pyridine via breast milk or directly induces intestinal tumors in multiple intestinal neoplasia mice Carcinogenesis 1999 20 1277 1282 10.1093/carcin/20.7.1277 10383901

