# Supplementary material for: Protein kinase A antagonist inhibits β-catenin nuclear translocation, c-Myc and COX-2 expression and tumor promotion in ApcMin/+ mice
Source: Mol Cancer. 2011 Dec 15;10:149. doi: 10.1186/1476-4598-10-149 (PMC3278393; doi:10.1186/1476-4598-10-149)
Supplement: Additional file 1 — Supplementary information. Materials and Methods. Reference List [27-29]. [file 1476-4598-10-149-S1.DOC]

**SUPPLEMENTARY INFORMATION**

**to**

**Brudvik et al.** *Protein kinase A antagonist inhibits β-catenin nuclear translocation, c-Myc and COX-2 expression and tumor promotion in Apc^Min/+^ mice*

**Materials and Methods**

Six-week old, female *Apc*^Min/+^ mice (PCR-genotyped) obtained by breeding C57BL/6J wild-type (*Apc*^+/+^) females with C57BL/6J *Apc*^Min/+^ males (Jackson Laboratory, Bar Harbor, ME) were randomized into three groups of 10 animals and treated with Rp-8-Br-cAMPS (Rp-8-Br; 75 mg/kg, prepared by Lauras AS (Oslo, Norway) [27], previously dose and toxicity tested [28], injected i.p. every second day), indomethacin (Indo; 5 mg/kg in ethanol; Sigma-Aldrich, MO, USA, delivered by gavage every second day [29]) or PBS (vehicle control , injected i.p. every second day) for 6 weeks (standard chow and maintenance, Norwegian Institute of Public Health), sacrificed and intestines subsequently examined. Two animals in the Rp-8-Br-cAMPS group were euthanized during the first 3 weeks due to rectal prolapse; two animals, one each from the Rp-8-Br-cAMPS and control groups, were excluded as outliers due to tumor numbers > mean + 3SD.

Small and large intestines were cut longitudinally, spread on filter paper, fixed (10% formalin, 48h), stained with 0.2% methylene blue in 10% formalin and examined by transillumination in an inverse light microscope (320x; blinded data acquisition), as described in detail elsewhere [30]. The study was approved by the National Animal Research Authority (id: FOTS1597) before conducting the experiments. We fully complied with the guidelines issued and exercised due consideration. The health of the animals was checked daily.

Immunohistochemistry was performed on sections from the distal third part of the small intestine and tumors were analyzed from all animals in each group. Formalin fixed intestines were rolled (Fig. 1), embedded in paraffin, subjected to serial sectioning at 3.5 µm and stained routinely with hematoxylin and eosin (H-E) or subjected to immunohistochemistry (Lab Vision Autostainer 480, AH-Diagnostics, Denmark) using antibodies to CD3 (Abcam, UK; 0.5 µg/ml), Granzyme B (Abcam, UK; 2.5 µg/ml), CD8 (Santa Cruz, CA, USA; 0.025 µg/ml), CD56 (Abcam, UK; 5 µg/ml), FOXP3 (Aviva Systems Biology, CA, USA; 1.67 µg/ml), HIF-1α (Novus Biologicals, CO, USA; 1.67 µg/ml), β-catenin (Thermo Fischer Scientific, CA, USA; 0.8 µg/ml), c-Myc (Santa Cruz, CA, USA; 4 µg/ml) and COX-2 (Thermo Fischer Scientific, CA, USA; 0.25 µg/ml) followed by blocking with endogenous peroxidase and staining with horseradish peroxidase, then examination in a DM 3000 microscope with DFC 420 camera (Leica, Germany).

Images captured with a 10x objective were analyzed using Image J 1.43u software package (National Institutes of Health, USA). On average 10 sections from each animal were created and a total of 1014 images were captured and analyzed (CD3; 138, CD8; 76, CD56; 96, GZMB; 155, FOXP3; 100, b-Catenin; 139, COX-2; 161, cMyc; 69 and HIF-1a; 80). The intensity of the HIF-1α, β-catenin and COX-2 staining were calculated as the mean inverse grayscale intensity of tumor area: The area of the lesions was marked with the Freehand Selection tool and then analyzed by the measure tool to calculate the mean grayscale intensity in the area. The mean grayscale was then inverted on the grayscale intensity scale (1-255). To exclude the possibility of bias because of background staining, the normal mucosa was evaluated using the same method with no differences observed between the treatment groups. Furthermore, the size of the tumor area used for grayscale calculations was registered, but there was no correlation between tumor number or tumor size and the staining intensity (Spearman Correlations R < 0.16, P > 0.05). Moreover, a direct coupling between observations grayscale intensity (i.e. β-catenin levels) and tumor size was precluded by the experimental setup where immunohistochemistry was done on sections (which may not represent the true size of the tumor) whereas the tumor size observations came from examinations in an inverse light microscope directly on whole mice intestines. SigmaPlot 11.0 (CA, USA) was used to compare groups and create graphs. A *P-*value < 0.05 was regarded significant. Median values were compared with Mann-Whitney Rank Sum test when Shapiro-Wilk Normality test failed; otherwise mean values were compared by Student’s *t*-test. Animals with tumor numbers outside three standard deviations from the mean of the group were excluded when comparing the groups.

Western blotting was performed on cell lysates from HCT 116 colorectal carcinoma cell line (ATCC, VA, USA) after stimulation (PGE_2_) in the absence or presence of inhibitors (indomethacin or Rp-8-Br-cAMPS) for different periods of time (30 min., 1 hour and 2 hours). The following antibodies were used β-catenin, phospho-β-catenin (Ser552), phospho-β-catenin (Ser675), (Cell Signaling Technology, MA, USA), c-Myc and Actin (Santa Cruz, CA, USA). Cell lysates and were analyzed on a 10% SDS/PAGE and blotted onto PVDF membranes. The filters were blocked in 5% non-fat dry milk in TBST or 5% BSA (according to manufacturers protocol) for 30 min at RT, incubated overnight at 4°C with primary antibodies, washed five times 5 min in TBST and incubated with a horseradish-peroxidase-conjugated secondary antibody. Blots were developed by using Supersignal West Pico substrate (Pierce, Rockford, IL, USA) and exposure of film and Image J software was next used for quantification of the immunoreactive signals.
