# Supplementary figures and images for: Protein kinase A antagonist inhibits β-catenin nuclear translocation, c-Myc and COX-2 expression and tumor promotion in ApcMin/+ mice
Source: Mol Cancer. 2011 Dec 15;10:149. doi: 10.1186/1476-4598-10-149 (PMC3278393; doi:10.1186/1476-4598-10-149)

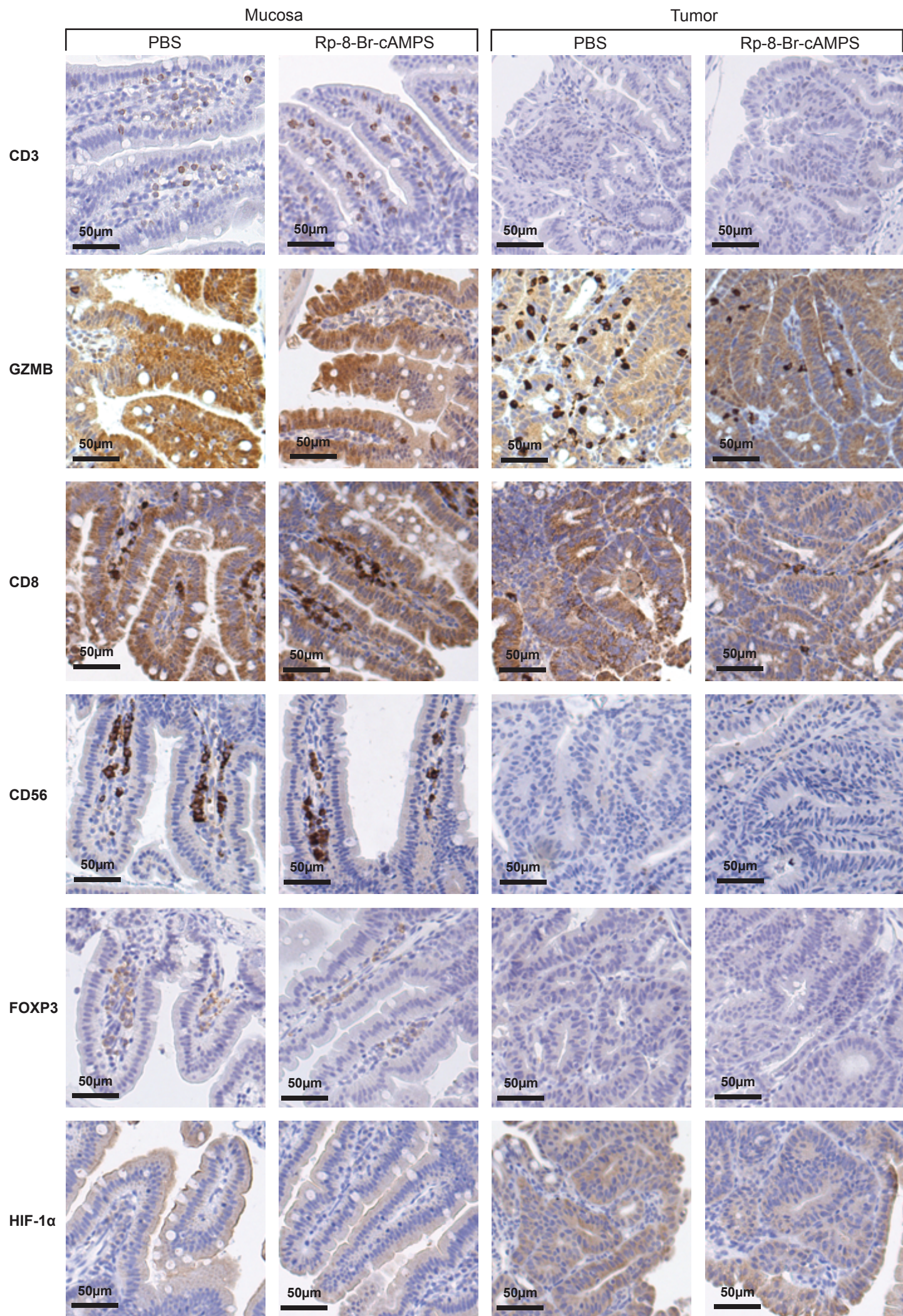

Fig. S1 - Brudvik et al.

Supplement: Additional file 2 — Figure S1. Immunohistochemical staining with indicated antibodies of tumor and normal mucosa from small intestines from ApcMin/+ mice treated with PBS or Rp-8-Br-cAMPS. [file 1476-4598-10-149-S2.PDF]

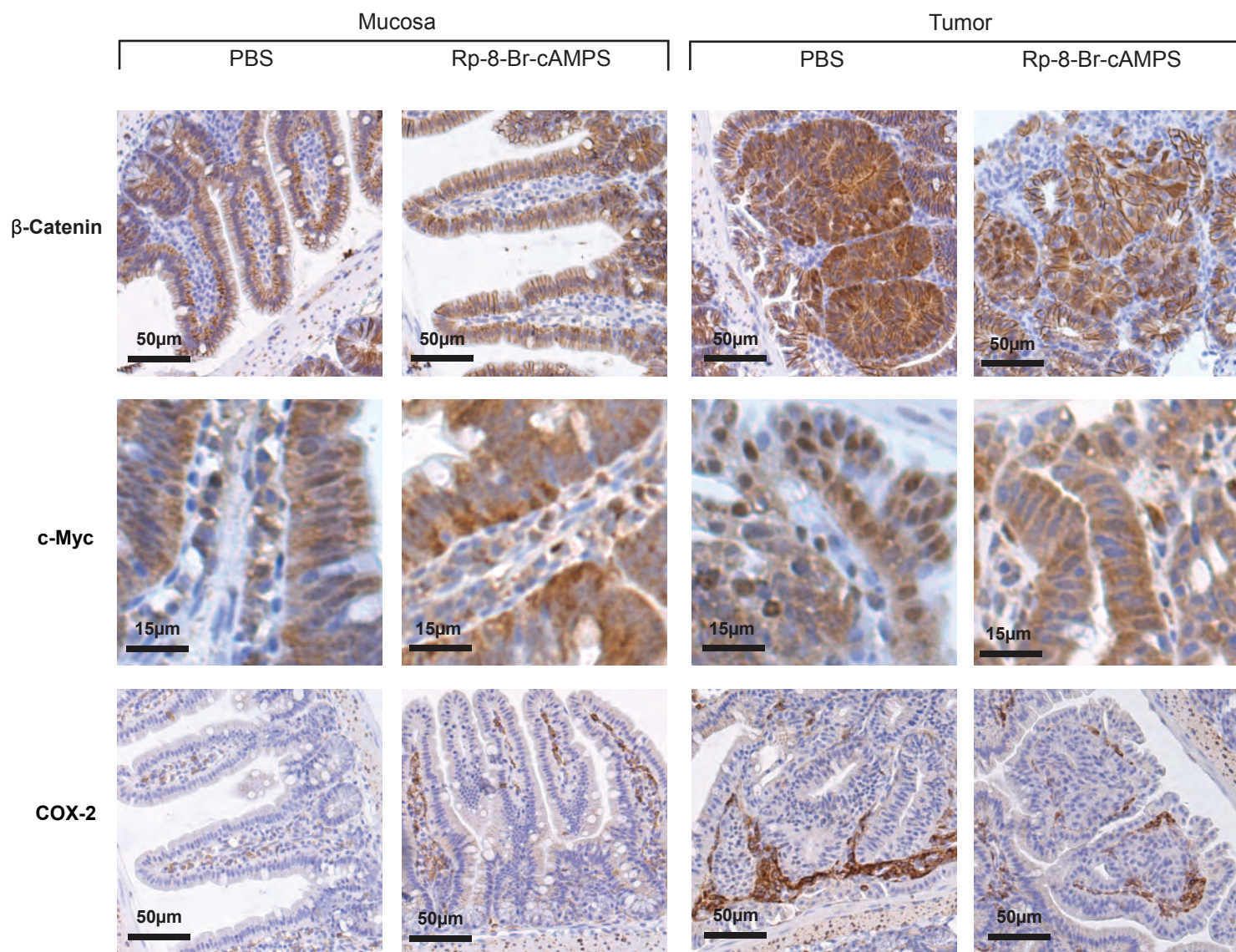

Fig. S2 - Brudvik et al.

Supplement: Additional file 3 — Figure S2. Immunohistochemical staining with indicated antibodies of tumor and normal mucosa from small intestines from ApcMin/+ mice treated with PBS or Rp-8-Br-cAMPS. [file 1476-4598-10-149-S3.PDF]
